# Parental smoking and cessation during pregnancy and the risk of childhood asthma

**DOI:** 10.1186/s12889-016-3029-6

**Published:** 2016-05-24

**Authors:** Maijakaisa Harju, Leea Keski-Nisula, Leena Georgiadis, Seppo Heinonen

**Affiliations:** Department of Obstetrics and Gynecology, Kuopio University Hospital, P.O. Box, 100, FI-70029 Kuopio, Finland; University of Eastern Finland, Faculty of Health Sciences, Kuopio, Finland; Department of Environmental Health, National Institute for Health and Welfare (THL), Kuopio, Finland; Department of Obstetrics and Gynecology, Helsinki, Finland; University Central Hospital, Helsinki, Finland

**Keywords:** Smoking, Pregnancy, Childhood, Asthma, Maternal, Paternal, Parental

## Abstract

**Background:**

To evaluate the association between maternal and paternal smoking during pregnancy, and asthma among offspring.

**Methods:**

We conducted a hospital-based birth retrospective observational birth cohort study in a University-based Obstetrics and Gynecology Department, Kuopio University Hospital, Finland. 39 306 women, delivering between 1989 and 2006, were linked to the national register for asthma reimbursement for their offspring (2641 asthmatics). Pregnancy factors were recorded during pregnancy.

**Results:**

The risk of asthma was significantly elevated if both parents smoked (aOR 3.7; 95 % Cl 3.2-4.4) and it remained high in only paternal smoking families (aOR 2.9; 95 % Cl 2.5-3.3) as well as only maternal smoking families (aOR 1.7; 95 % Cl 1.2-2.2). Paternal cessation of smoking during pregnancy seemed to reduce the risk of asthma regardless of maternal smoking (aOR 0.3-0.4).

**Conclusions:**

Parental smoking, and especially paternal smoking, was significantly associated with the risk of asthma in offspring and paternal cessation of smoking during pregnancy was associated with a decreased risk of childhood asthma regardless of maternal smoking. The results indicate that both parents should be encouraged to quit smoking during pregnancy, since it is a relatively easy and cheap way to reduce the risk of asthma in offspring.

**Trial registration:**

The study is registered in Kuopio University Hospital register (TUTKI): ID5302448

## Background

Asthmatic diseases are among the most common chronic diseases affecting both children and young adults [1–3]. The prevalence of asthma among children varies worldwide, being lowest, only 2-4 %, in Asian countries and highest in the United Kingdom, Canada, Australia and other developed countries, where the prevalence might be up to 15 to 20 % [1, 2]. In Finland, the prevalence of asthma among children varies between 7 to 9 %, but has diminished about 30 % in the past decade especially among children aged 0–4 years of age probably because of more specific diagnostic criteria [4]. The etiology and risk factors are often categorized to genetic, environmental and host factors, and global differences are probably a result of gene-by-environment interactions [2, 3].

The prenatal risk factors of asthma are multifactorial, including maternal smoking (both active and passive), maternal stress and exposure to various substances such as antibiotics, dietary interventions during pregnancy, and birth by cesarean section [3, 5, 6]. Maternal smoking also affects fetal growth, increasing the risks of low birth weight infants and preterm birth, which in turn are also known to increase the risk of asthma [5, 7–9]. Both maternal prenatal smoking and postnatal exposure to environmental tobacco smoke (ETS) are associated with elevated risks of behavioural problems, neurocognitive deficits and sudden infant death syndrome [9, 10].

Pregnancy is known to be a critical period of developmental programming, where maternal smoking might influence fetal immune development through activating or silencing fetal genes, affecting lung growth and differentiation, and predisposing offspring to asthma. Exposure to ETS during pregnancy is also associated with the same effects and, through genetic variation, a proportion of the population has been shown to be especially vulnerable to such exposure [1, 3, 11, 12].

There are only a few reports on the association between different patterns of parental smoking, especially paternal smoking during pregnancy, and asthma among offspring. Håberg et al. found increased risk of wheezing in families with postnatal exposure to paternal smoking independently of maternal smoking during pregnancy, but not prenatally [1]. Burke et al. (2012) published a large review article that revealed that exposure to maternal smoking, in particular during prenatal or postnatal period, was associated with significantly increased risks of onset of asthma and wheeze in children. Data on the effect of exposure to paternal postnatal smoking were limited since they found only two studies with significant results out of five studies included in meta-analysis that revealed a significantly increased risk of incidence of asthma before the age of 11 years [13]. Therefore, we assessed the effects of different parental smoking patterns during pregnancy in connection with the risk of asthma in childhood.

## Methods

The study was a retrospective hospital-based birth cohort study and the data was derived from the clinical birth database of the Department of Obstetrics and Gynecology, Kuopio University Hospital, and the Social Security Institution of Finland register for asthma medication reimbursement. Data collected between January 1989 and December 2008 covered 45 030 pregnant women and their infants. After exclusion of stillbirths (*n* = 175), neonatal deaths (*n* = 154), infants born before 23 weeks of gestation (*n* = 20), children born 2007–2008 (*n* = 4867) and missing information (*n* = 487), the data covered 39 306 women with live-born infants. In this cohort, 2641 children had asthma medication. Parental (maternal/paternal) smoking was evaluated as: non-smoker, smoker (daily smoking before and during pregnancy) and a quitter (quit smoking before the onset of pregnancy or before 13 + 0 GWs). Information on maternal and paternal pre-pregnancy characteristics was based on maternally reported data collected from self-administered questionnaires at 20 weeks of pregnancy. Missing data were added by midwives and nurses during interviews at visits to prenatal maternity clinics or labor wards at Kuopio University Hospital. The questionnaire consisted of 75 questions on background items such as health habits, including both maternal and paternal smoking, previous pregnancies and chronic diseases. Information on pregnancy complications, pregnancy outcome and the neonatal period was added by midwives and nurses at the time of delivery and during the neonatal period. Informed consent for this register study was given by childbearing women at the time of data collection.

Information on the need of anti-asthmatic drugs during childhood was obtained from the Drug Prescription Register of the Social Insurance Institution between years 1989–2008. All medication in Finland is registered in the Drug Prescription Register. The cost of medication prescribed by physicians, and in the case of pediatric asthma, by pediatricians, is reimbursed by 72 % through the National Sickness Insurance Scheme. Anti-asthmatic drugs were categorized according to ATC-groups R06 e.g. inhaled glucocorticoids, inhaled long acting beta2-agonists and antileukotrienes. In the present study, children who were entitled to a special reimbursement for anti-asthmatic drugs and purchased them at least once after the diagnosis were considered as asthmatics [8, 14].

The parameters of variables were tabulated and differences between asthmatics and non-asthmatics were tested by using Chi-square and Mann–Whitney *U* tests for statistical significance. Logistic regression analyses were used to investigate the multivariate-adjusted association between parental smoking and asthma diagnosed among offspring. Analyses were performed using SPSS 21.0 for Windows software.

We adjusted the analyses for maternal asthma (no vs. yes), maternal age (≤25, 26–35, ≥36 years), maternal parity (0, 1, ≥2), pre-pregnancy BMI (<21, 21–25, >25 kg/m^2^), ART (assisted reproduction technology (no vs. yes)), marital status (not married vs. married), child’s sex, gestational weeks at birth (<37, 37–40, ≥ 41) and mode of delivery (vaginal vs. cesarean). Furthermore, a child’s follow-up time was taken into account by using the child’s age at the onset of asthma, or among healthy children, age at the end of the study (2006). A p-value of <0.05 was deemed to be statistically significant.

To examine whether background information (ie, maternal age, asthma, marital status, mode of delivery, gestational weeks at birth and gender of child) contributed to the differences in childhood asthma incidence related to parental smoking, we estimated the contribution of each of these factors adding them separately to logistic regression analysis.

## Results

Table 1 shows the characteristics of the study population. Among all 39 306 live-born children, 2641 (6.7 %) had asthma medication. 70.6 % of children with asthma were followed over 10 years (data not shown) and the mean follow-up time was 4.2 years. All children were over two years of age and more than 80 % of children had asthma by the age of seven years. Asthmatic children were more frequently males and born to mothers with asthma and hypertension. They were also born more frequently to mothers with earlier infertility problems and to married couples. Children with asthma were born at earlier gestational weeks, more often by cesarean section and with lower birth weight and lower Apgar scores compared with non-asthmatics.

**Table 1:** Basic characteristics of the study population in relation to asthma at any time among offspring

	Asthma*	No asthma*	*p*-value**
	*n* = 2641 (6.7 %)	*n* = 36 665 (93.3 %)	
Primipara	1 039 (39.3)	15 131 (41.3)	0.114
Maternal			
Asthma	116 (4.4)	876 (2.4)	<0.001
GDM^a^	143 (5.4)	2 314 (6.3)	0.066
Hypertension	76 (2.9)	725 (2.0)	0.002
Preeclampsia	115 (4.4)	1 438 (3.9)	0.217
ART^b^	183 (6.9)	1736 (4.7)	<0.001
Maternal age, years	28.7 (5.3)	29 (5.5)	0.313
<25	780 (29.5)	9 944 (27.1)	
26-35	1 581 (59.9)	22 049 (60.1)	
>35	280 (10.6)	4672 (12.7)	0.001
Mean prepregnancy BMI^c^, kg/m^2^	23.4 (4.5)	23.2 (4.3)	0.020
Mean number of previous pregnancies	1.5 (1.6)	1.4 (1.6)	0.08
Mean number of previous deliveries	1.1 (1.2)	1.0 (1.3)	0.57
Marital status			
not married	900 (34.1)	13 238 (36.1)	
married	1 741 (65.9)	23 427 (63.9)	0.036
Mode of delivery			
Vaginal	2 040 (77.2)	29 925 (81.6)	
Cesarean section	601 (22.8)	6 740 (18.4)	<0.001
Single	2 500 (94.7)	35 340 (96.4)	
Twins	133 (5.0)	1 258 (3.4)	
Triplets	8 (0.3)	67 (0.2)	<0.001
Premature delivery	380 (14.4)	2 594 (7.1)	<0.001
Boy	1 602 (60.7)	18 241 (50.2)	<0.001
Mean gestational age, w	38.4 (3.2)	39.2 (2.1)	<0.001
Apgar score 5 min <7	70 (2.7)	663 (1.8)	0.002
Chorioamnionitis^d^	48 (1.8)	414 (1.1)	0.002
Mean follow-up time of children, years*	4.2 (3.6)	9.3 (5.3)	<0.001

*Values are mean (±2 SD) or N (%)

** p-value obtained in univariate model

^a^Gestational diabetes mellitus

^b^ART (assisted reprodution technology), including IVF (in-vitro fertilization), ICSI (intracytoplasmic sperm injection), insemination and ovulation induction by clomiphene and other medicines

^c^Data missing in 1984 (4.5 %) cases

^d^Data missing in 7 (0 %) cases

Table 2 shows the differences in parental smoking habits in relation to healthy and asthmatic offspring. The proportion of cessation of maternal smoking before or during pregnancy was similar in both groups (13.0 %), but children with asthma significantly more often had smoking mothers and/or fathers throughout the pregnancy compared with children without asthma (mothers: 14.0; fathers: 29.5 %). Pregnancy little affected paternal smoking habits: 24.5 % of fathers continued smoking throughout the pregnancy Asthma prevalence was highest among families where either the mother or father smoked (7.8 % and 8.1 %). Isolated paternal smoking increased the risk of asthma 2.7–fold and isolated maternal smoking increased it 1.4–fold compared with the risk associated with non-smoking parents. In our study population maternal smoking during pregnancy was decreased from 13.2 % to 9.6 % in a ten year period but similar trend in the proportion of cessation was not seen among mothers (Data not shown).

**Table 2:** Parental smoking characteristics before and during pregnancy

				Asthma			
	Total	No asthma		Prevalence			
	N (%)	N (%)	Asthma n (%)	n/N (%)	*p*-value	aOR* 95%CI	*p*-value**
Maternal							
Non-smokers^a^	28743 (73.1)	26881 (73.3)	1862 (70.5)	6.5		1	<0.001
Recent quitters^b^	5103 (13.0)	4755 (13.0)	348 (13.2)	6.8		1.10 (1.0-1.3)	0.218
Smokers	4748 (12.1)	4379 (11.9)	369 (14.0)	7.8	0.001	1.36 (1.2-1.6)	<0.001
Missing	712 (1.8)	650 (1.8)	62 (2.3)	8.7			
Paternal							
Non-smokers^a^	22394 (57.0)	20921 (57.1)	1473 (55.8)	6.6		1	<0.001
Recent qutters^b^	4763 (12.1)	4528 (12.3)	235 (8.9)	4.9		0.31 (0.3-0.4)	<0.001
Smokers	9633 (24.5)	8853 (24.1)	780 (29.5)	8.1	<0.001	2.73 (2.4-3.1)	<0.001
Missing	2516 (6.4)	2363 (6.4)	153 (5.8)	6.1			

^a^No smoking before or during pregnancy

^b^Quit smoking prior to or during pregnancy

*Adjusted for the following maternal factors at birth: asthma, age, parity, pre-pregnancy BMI, ART, and marital status, parental smoking plus gender of child, gestational weeks at birth, mode of delivery and age at the onset of asthma, or among controls, follow-up age

**p-value for adjusted analysis obtained from the trend test (Wald) in logistic regression analysis

We further evaluated the significance of different parental smoking habits during pregnancy by multivariate analysis (Table 3). The risk of asthma was increased in all cases with paternal smoking, regardless of maternal smoking. The risk of asthma was highest among children when both parents smoked (aOR 3.7, 95 % Cl 3.2-4.4) compared with children of non-smoking parents. The risk of asthma was also significantly increased in families when either one of the parents smoked (mother: aOR 1.7, 95 % Cl 1.2-2.2, father: aOR 2.9, 95 % Cl 2.5-3.3) compared with children of non-smoking parents. Among families where the father smoked and the mother quit during pregnancy, the risk of asthma among children was still significantly increased (aOR 2.8, 95 % Cl 2.3-3.4). The interaction testing between maternal and paternal smoking status were non- significant (*p* = 0.727). However, a protective effect as regards the risk of asthma among offspring was detected in all cases when the father quit smoking in early pregnancy, regardless of maternal smoking (aORs 0.3-0.4) (Table 3).

**Table 3:** Association between parental smoking during pregnancy and risk of asthma at any time among offspring

Maternal and paternalsmoking	No asthma *N* = 36665	Asthma *N* = 2641	Asthma Prevalence (%)	OR 95%CI	*p*-value	aOR*95%CI	*p*-value**
No maternal smoking							
Paternal non-smoker^a^	18828 (51.4)	1307 (49.5)	6.5	1		1	
Paternal recent quitter^b^	2362 (6.4)	124 (4.7)	5.0	0.76 (0.6-0.9)	0.004	0.35 (0.3-0.4)	<0.001
Paternal smoking	4463 (12.2)	370 (14.0)	7.7	1.19 (1.1-1.3)	0.004	2.87 (2.5-3.3)	<.0.001
Maternal recent quitter^b^							
Paternal non-smoker^a^	1271 (3.5)	98 (3.7)	7.2	1.11 (0.9-1.4)	0.334	1.17 (0.9-1.5)	0.182
Paternal recent quitter^b^	1278 (3.5)	68 (2.6)	5.1	0.77 (0.6-1.0)	0.337	0.41 (0.3-0.5)	<0.001
Paternal smoking	1966 (5.4)	176 (6.7)	8.2	1.29 (1.1-1.5)	0.002	2.81 (2.3-3.4)	<0.001
Maternal smoking							
Paternal non-smoker^a^	776 (2.1)	65 (2.5)	7.7	1.21 (0.9-1.6)	0.156	1.65 (1.2-2.2)	0.001
Paternal recent quitter^b^	883 (2.4)	43 (1.6)	4.6	0.70 (0.5-1.0)	0.026	0.30 (0.2-0.4)	<0.001
Paternal smoking	2378 (6.5)	234 (8.9)	9.0	1.42 (1.2-1.6)	<0.001	3.74 (3.2-4.4)	<0.001
Missing data	2460 (6.7)	156 (5.9)	6.0				

^a^No smoking before or during pregnancy

^b^Quit smoking prior to or during pregnancy

*Adjusted for the following maternal factors at birth: asthma, age, parity, pre-pregnancy BMI, ART, and marital status, plus gender of child, gestational weeks at birth, mode of delivery and age of asthma or among controls follow-up time

**p-value for adjusted analysis obtained from the trend (Wald) in logistic regression model

Since maternal exposure to paternal smoking might be more direct in married families, we evaluated separately children with married couples (Table 4). In this subgroup analysis, no significant differences were observed.

**Table 4:** Association between parental smoking during pregnancy and risk of asthma at any time among offspring in married families

	No asthma *N* = 23427	Asthma *N* = 1741	Asthma prevalence (%)	aOR*95%CI	*p*-value**
No maternal smoking					
Paternal non-smoker^a^	14227 (60.7)	1040 (59.7)	6.8	1	
Paternal recent quitter^b^	1228 (5.2)	64 (3.7)	5.0	0.30 (0.2-0.4)	<0.001
Paternal smoking	2967 (12.7)	251 (14.4)	7.8	3.01 (2.6-3.7)	<.0.001
Maternal recent quitter^b^					
Paternal non-smoker^a^	679 (2.9)	51 (2.9)	7.8	1.12 (0.8-1.5)	0.0483
Paternal recent quitter^b^	528 (2.3)	35 (2.0)	6.2	0.50 (0.3-0.7)	<0.001
Paternal smoking	1008 (4.3)	87 (5.0)	7.9	2.71 (2.1-3.5)	<.0.001
Maternal smoking					
Paternal non-smoker^a^	323 (1.4)	27 (1.6)	7.7	1.59 (1.0-2.5)	0.042
Paternal recent quitter^b^	280 (1.2)	13 (0.7)	4.4	0.22 (0.1-0.4)	<0.001
Paternal smoking	966 (4.1)	101 (5.8)	9.5	3.94 (3.1-5.1)	<0.001
Missing data	1221 (5.2)	72 (4.1)	5.6		

^a^No smoking before or during pregnancy

^b^Quit smoking prior to or during pregnancy

*Adjusted for the following maternal factors at birth: asthma, age, parity, pre-pregnancy BMI and ART, plus gender of child, gestational weeks at birth, mode of delivery and age of asthma or among controls follow-up time

**p-value for adjusted analysis obtained from the trend test (Wald) in logistic regression model

## Discussion

Our main finding was that parental smoking, and especially paternal smoking regardless of maternal smoking, increased the risk of asthma among offspring. We further observed that the risk was cumulative in nature, since maternal smoking increased the risk of asthma 1.7-fold compared with non-smokers, paternal smoking increased it 2.9-fold and when both parents smoked there was a 3.7-fold increased risk. Interestingly, paternal smoking represented an even more significant risk of asthma among offspring than maternal smoking. Several reasons might explain these findings. First, because of changes in the social acceptability of smoking and reports on the possible adverse consequences of smoking, mothers might have been more inclined to deny or underestimate their smoking. Further, there is some evidence that prenatal exposure might have a greater impact on pediatric asthma than postnatal exposure [15]. However, the precise effect of pre- and postnatal exposure to ETS on childhood asthma and other adverse outcomes are difficult to identify, since it is likely that women who smoke during pregnancy continue smoking after delivery [16]. Furthermore, ETS itself is difficult to measure, since smoking outdoors reduces but does not completely protect children from ETS exposure at home [17]. It is estimated that about 50 % of women who quit smoking just before or during pregnancy might smoke again after delivery [16, 18]. Therefore, excessive effect of paternal smoking to childhood asthma might be due to mothers who quit smoking, but continued it after delivery and thus exposed their offspring to ETS.

Maternal cessation reduced the risk only from 3.7- to 2.8-fold, and surprisingly, paternal smoking cessation during pregnancy showed an even more protective effect against asthma in offspring regardless of maternal smoking. One of the potential biological mechanisms of paternal smoking and childhood asthma is that the mother and the fetus were passively exposed to smoking. On the other hand, one would assume that a true causal effect like a programming effect from prenatal smoking exposure would be higher when directly exposed through maternal smoking, compared with being indirectly exposed through paternal smoking. Accordingly it is well known that smoking habit is a marker of risky healthy behavior and therefore those who continue smoking may represent a high risk group causing a residual confounding and some bias to the study setting.

Paternal cessation of smoking during pregnancy reduced the risk of asthma among offspring 0.4-fold. The prevalence of asthma was low (4.6 to 5.1 %) if the father was a recent quitter. It was intriguing how fathers who quit smoking during pregnancy had lower risk of asthma in the offspring compared to non-smoking fathers. It has been shown in two earlier studies that paternal smoking after birth is associated with an increased risk of respiratory diseases among offspring, but prenatal paternal smoking was found not to have such an effect [1, 13]. However, earlier studies have not been focused on the significance of paternal smoking or cessation during pregnancy and the risk of asthma among offspring. Our findings confirm the previously shown well-known increased risk of asthma among offspring with maternal prenatal smoking [1, 5, 9, 19]. However, such a findings might have been due to the “healthy smoker” effect, where reduced postnatal ETS might have affected the results, since the true effect of prenatal paternal smoking might has been small [19].

Further, we found that maternal cessation of smoking had a protective effect against asthma, as shown before [9]. The incidence of asthma was reduced from 7.7 % to 7.2 % if the mother quit, and if both parents quit smoking, asthma prevalence diminished even more-from 9.0 to 5.1 %. Reasons for smoking and cessation are multifactorial. Maternal smoking during pregnancy has been associated with fear of weight gain, partners’ or friends’ smoking habits and psychiatric comorbidities. Smoking cessation is more common in parents with higher socioeconomic status, low-level cigarette smoking before pregnancy, awareness of the effects of smoking during pregnancy and in parents who are more responsible towards children and family [20, 21]. Parents, and especially fathers, who quit smoking before the childbirth were possibly more likely to be nonsmokers after birth, they probably shielded their offspring from secondhand exposure to smoke and were more likely to stop smoking when children exhibited signs of respiratory morbidity in early life.

The prevalence of maternal smoking during pregnancy varies between countries, but in industrialized countries its incidence has varied between 13 and 38 % in the latest reports [9]. Prenatal exposure to smoking is reported to be less than 7 % in some Eastern countries, but higher than 25 % in Holland, Poland and North America [19]. In Finland, the incidence of the maternal smoking is 16 % and has been at the same level as in the late 1980s. In 2012, about 17 % of Finns aged 15–64 smoked daily and accounted for 21 % of men and 14 % of women [22, 23]. Our results are comparable, varying from 12.1 % among pregnant women to 24.5 % among their spouses during pregnancy. Smoking during pregnancy is known to be more prevalent among younger and less well-educated women and those with low incomes [21, 24]. Smoking mothers also more often have unintended pregnancies and they smoke more cigarettes before pregnancy compared with those who quit. Being unmarried is also known to be associated with smoking during pregnancy [16, 20, 24]. Our results are similar, since smoking mothers were younger (more frequently less than 25 years old) and more frequently unmarried (data not shown).

The most significant strength of this study is the quality of the data. We gathered data from a single university hospital with uniform collection criteria. The personal identity code system used in Finland gave us the opportunity to link our birth register database to the register at KELA, the Social Insurance Institution of Finland. This institution gave us reliable data on pediatric asthma, as evaluated similarly in two earlier Finnish studies concerning childhood asthma [8, 14]. We also managed to control a relatively large number of potential confounding factors known to affect asthma risk among offspring. On the other hand, data has been produced mainly for administrative and statistical purposes rather than for research and cannot be used to indicate causality in the study, only the association between parental smoking and asthma among offspring.

Our study has some limitations. Unfortunately we had no precise information on the amount of cigarettes smoked per day and therefore were not able to examine a possible dose–response aspect of the risk of childhood asthma. This would have been interesting, since maternal smoking has been shown to be a dose-dependent risk factor as regards the development of asthma [25, 26]. Further, there is not a precise definition of smoking and smoking cessation during pregnancy in epidemiological studies. In at least two previous studies the definition of smoking has been at least one cigarette per day [27, 28]. Therefore, our classification might have resulted in underestimation of the actual number of smokers. Further, self-reported smoking is considered to be a valid way to study tobacco exposure during pregnancy in observational studies in comparison with assessment of biomarkers such as cotinine [29]. However, it can be argued that self-reporting may has led to underestimation of the prevalence of smoking [30], even though our data was based on both antenatal self-reporting and maternity case notes.

In this study we could not use marital status as a definite proxy for father living with pregnant spouse or not since cohabiting couples (unmarried) are also mainly living in same household. It is very popular and common phenomenon in Finland living together without marriage nowadays. However, marital status was included as confounders since outcome variable- childhood asthma and parental smoking is very much related to marital status as well- that is cohabiting parents do smoke more often compared to married parents. An additional sub-analysis did not reveal any stronger association in married families.

Smoking is known to be a marker of lifestyle and health behavior and is often associated with social status. Unfortunately we had no information on SES and causality cannot therefore be shown in this kind of study setting; only conclusions about associations can be suggested, taking the known weaknesses of register-based studies into account. We also lack information on the environmental risk factors of asthma, such as pollution, daycare attendance, socio-economic markers and neonatal respiratory morbidity after birth. Furthermore, we chose logistic regression analyses to investigate the multivariate-adjusted risk of asthma among offspring. Children’s follow-up times represented a variable that might have brought some bias to the results and children who developed asthma at a younger age might have had greater weight in analysis.

## Conclusion

Smoking during pregnancy is associated with many pregnancy complications such as preterm delivery, low birth weight, placental abruption, and placental insufficiency [9, 10, 21, 31, 32]. We showed here how parental smoking, and especially paternal smoking, has important effects on the incidence of asthma among offspring. This should encourage healthcare professionals to support both parents in quitting smoking during pregnancy in order to reduce the risk of asthma among offspring. Smoking cessation during pregnancy is a relatively easy and cheap way to reduce asthma morbidity among children.

### Ethics

The study was approved by Ethics Research Committee of Kuopio University Hospital and the Committee gave permission for the results to be published (93//2008).

### Consent for publication

Not applicable.

### Availability of data and materials

The dataset supporting the conclusions of this article is not available since Ethics Research Committee of Kuopio University Hospital and the Committee gave permission to use material only for researchers (93//2008). Therefore, this kind of register-based research data can never be released for general public due to confidential and privacy issues.

## Abbreviations

aOR: adjusted odds ratio
Cl: confidence interval
ETS: environmental tobacco smoke
